# Incidence and Predictors of Pulmonary Embolism Recurrence and Mortality in Patients With Lung Cancers

**DOI:** 10.7759/cureus.93937

**Published:** 2025-10-06

**Authors:** Reem F Almesfir, Nada F Alqubaibi, Ahmed Alkhelaifi, Abdel Galil Abdel Gader, Fahad A Aleidan

**Affiliations:** 1 Medical Oncology, King Abdulaziz Medical City, Health Affairs, Ministry of National Guard, Riyadh, SAU; 2 Obstetrics and Gynaecology, King Faisal Specialist Hospital and Research Centre, Riyadh, SAU; 3 Orthopedics, King Abdulaziz Medical City, Health Affairs, Ministry of National Guard, Riyadh, SAU; 4 Medicine, King Saud Bin Abdulaziz University for Health Sciences, Riyadh, SAU

**Keywords:** d-dimer, lung cancer, predictors, pulmonary embolism, serum albumin

## Abstract

Background

Pulmonary embolism (PE) is a serious and potentially fatal complication in lung cancer patients. This study aims to identify predictors of recurrent PE and related mortality in this population.

Methods

A retrospective review was conducted using electronic medical records from King Abdulaziz Medical Cities in Riyadh and Jeddah, Saudi Arabia. Lung cancer patients with confirmed initial PE were included. Data on demographics, clinical features, and laboratory values-including Eastern Cooperative Oncology Group (ECOG) performance status, systemic infections, serum albumin, and D-dimer levels-were collected. PE and its recurrence were confirmed by standard imaging techniques. The statistical analyses comprised descriptive statistics, analysis of the receiver operating characteristic (ROC) curve to establish the optimum cutoff values of the biomarkers, and logistic regression to identify predictors of PE recurrence and mortality.

Results

A total of 98 adult lung cancer patients with initial PE were identified. Of these, 19 (19.4%) experienced recurrent PE, with a mortality rate of 73.7% in this group. Recurrent PE was significantly associated with poor ECOG performance status and systemic infections. Laboratory findings showed that recurrent cases had lower serum albumin (≤ 20.5 mg/L) and higher D-dimer (≥ 6.5 mg/L) levels. Logistic regression revealed that adenocarcinoma type, systemic infection, elevated D-dimer, and low albumin were significant predictors of PE recurrence. Mortality was higher in the recurrence group compared to non-recurrence (73.7% vs. 44.3%, p = 0.021).

Conclusion

Recurrent PE occurs in 19.4% of lung cancer patients with initial PE and carries high mortality. Identified predictors warrant validation in larger studies.

## Introduction

Pulmonary embolism (PE) is one of the most frequently encountered severe complications in lung cancer patients and can have a significant effect on morbidity and mortality, resulting in poor outcomes and reduced survival [1]. Besides, a strong association has been reported between lung cancer and the risk of developing venous thromboembolism (VTE), a term that refers to both deep vein thrombosis and pulmonary embolism (PE) [2]. In lung cancer, PE can occur at a very high rate, with prevalence figures ranging from 3% to 20%, especially in patients with advanced or metastatic diseases [2]. However, in an earlier review of forty-one studies consisting of 13 randomized controlled trials, 27 retrospective cohorts, and one prospective cohort, PE has been reported in patients with lung cancer with an overall incidence of 3.7%, range: 0 to 23.7% [3]. 

It is now widely recognized that the classical risks for the formation of venous thrombosis are hypercoagulability, stasis, and vascular injury (the Virchow Triad) [4]. However, this understanding can only be partially applicable to VTE in lung cancer patients in whom hypercoagulability is a critical risk factor that increases the chances of PE. This is based on the fact that tumor cells can release pro-coagulant factors, especially tissue factor (TF) [5], which is now recognized as the major initiator of the clotting cascade in vitro. TF is a transmembrane glycoprotein, also known as thromboplastin, which is overexpressed on cancer cells [6]. TF enhances thrombin generation, increases the release of prothrombin fragment 1 + 2 (F 1 + 2; a marker of hemostatic activation and thrombin generation) and subsequent fibrin/clot formation, thereby predisposing patients to thrombotic events. In lung cancer patients, there are also additional prothrombotic factors in the form of elevated levels of plasma fibrinogen and platelet numbers [7,8]. Also, after chemotherapy, platelets display elevated PCA (procoagulant activity) and contribute further to the hypercoagulability in lung cancer [3]. 

Despite the availability of extensive information on lung cancer, there is still a need to improve the clinical outcome of these patients, and indeed, this has been the main trigger of our interest in the current study. Efforts to fulfill this goal depend on identifying predictors for both recurrent pulmonary embolism (PE) as well as mortality among lung cancer patients. Our attention will also focus on additional factors that contribute to and determine both recurrence of PE and total survival, mainly tumor histology, disease stage, as well as systemic therapy.

The primary aim of this study was to identify predictors of recurrent pulmonary embolism (PE) in patients with lung cancer, while a secondary, exploratory analysis examined overall mortality in this patient population and its association with clinical and laboratory variables. Accordingly, the study also aimed to identify the rates of PE recurrence in lung cancer patients and the critical determinants of mortality among these patients.

## Materials and methods

This is a retrospective cohort study that was conducted at King Abdulaziz Medical City (KAMC) in Riyadh and Jeddah, Saudi Arabia, in the period from January 1, 2016, to December 31, 2020. We included patients who were diagnosed with non-small cell lung cancer (NSCLC) or small cell lung cancer (SCLC) and had a history of PE confirmed by computed tomography (CT) after cancer diagnosis. The study included patients above the age of 18 years, who suffered one documented episode of PE, and were followed up for incidents of recurrence of PE or death till December 31, 2020, where recurrence of PE refers to any documented event during the follow-up period, irrespective of the exact timing.

Patients under 18 years of age and those who had no history of confirmed PE were excluded. Patients’ data was extracted from the electronic medical records (EMR), and included demographic information, such as age, sex, body mass index, smoking history, histological type of the lung cancer whether SCLC or NCSLC, clinical features, presence of metastasis, and comorbidities, such as diabetes mellitus, hypertension, dyslipidemia, chronic kidney disease, cardiac disease, liver disease, and obesity. Laboratory data were collected within 24 hours of confirmed recurrent PE, including serum albumin and D-dimer levels. Performance status assessments were made using the Eastern Cooperative Oncology Group (ECOG) scale, which ranges from 0 to 4 as follows: 0 = capacity to carry out daily living activities without restrictions as before diagnosis; 1 = restriction of strenuous physical activity with full ambulation and capacity to perform non-strenuous tasks; 2 = capacity to perform nonstrenuous self-care tasks without confinement to bed or chair for more than 50% of their waking hours; 3 = capacity to perform only limited self-care activities with confinement to bed or chair for more than 50% of their waking hours; and 4 = complete disability with confinement to bed or chair [9,10]. Poor ECOG scores were considered ≥ 2. 

Statistical analysis 

Descriptive statistics were used to summarize patient characteristics and laboratory findings. Continuous variables were compared using the Mann-Whitney U test, and categorical variables were compared using the chi-square test. Receiver operating characteristic (ROC) curve analysis was conducted to assess optimal cutoff values of serum albumin and D-dimer levels for recurrent PE, along with sensitivity, specificity, and the area under the curve with 95% confidence intervals. A logistic regression analysis was done to identify the predictors of PE recurrence and mortality, whose results were expressed in odds ratios and 95% confidence intervals (CI). All tests were done at a significance level of 0.05. All analyses were done using SPSS Statistics version 25.0 (IBM Corporation, Armonk, NY, USA).

## Results

Among the 98 lung cancer patients with initial pulmonary embolism (PE), 19 (19.4%) experienced PE recurrence, giving a recurrence rate of 19.4%. The mortality rate among patients with PE recurrence was 14 (73.7%), while the overall mortality rate for the cohort was 49 (50%), measured up to the end of December 2020. Demographic and characteristics of lung cancer patients with and without recurrence of PE are presented in Table 1. There were no differences between the recurrence and the no recurrence groups in the mean age, proportion of female sex, proportion of obesity, and smoking status. Furthermore, the following concurrent diseases: diabetes mellitus, hypertension, dyslipidemia, chronic kidney disease, cardiac disease, liver disease, obesity, and prior surgery, did not differ significantly between the two groups. However, the poor Eastern Cooperative Oncology Group (ECOG) performance status was significantly higher in the recurrence group [14 (73.7%) vs 34 (43.0%), p = 0.016], and systemic infection [14 (73.7%) vs 31 (39.2%), p = 0.007] was also significantly higher in the recurrence group. Similarly, the proportion of death was significantly higher in the recurrence group [14 (73.7%) vs 35 (44.3%), p = 0.021, Table 1].

**Table 1:** Demographics and characteristics ECOG: Eastern Cooperative Oncology Group; PS: performance status; SD: standard deviation.

Variables	Recurrence (n = 19)	No recurrence (n = 79)	p value
Age, years (±SD)	67.4 (±13.2)	65.5 (±13.9)	0.578
Female sex, no (%)	7 (36.8%)	28 (35.4)	0.909
Body mass index (BMI), kg/m^2^	23.8 (±8.2)	25.2 (±6.9)	0.507
Smoking	12 (63.2)	45 (57.0)	0.623
ECOG performance status (PS), no (%) Poor PS (ECOG ≥ 2)	14 (73.7)	34 (43.0)	0.016
Concurrent diseases, no (%)			
Diabetes mellitus	13 (68.4)	40 (50.6)	0.162
Hypertension	11 (57.9)	48 (60.8)0	0.819
Dyslipidemia	8 (42.1)	32 (40.5)	0.899
Chronic kidney disease	2 (10.5)	6 (7.6)	0.484
Cardiac disease	9 (47.3)	36 (45.6)	0.869
Liver disease	3 (15.8)	7 (8.9)	0.168
Risk factors, no (%)			
Obesity	5 (26,3)	23 (29.1)	0.808
Surgery	3 (15.8)	11 (13.9)	0.540
Systemic infection	14 (73.7)	31 (39.2)	0.007
Chemotherapy	16 (84)	55 (69.6)	0.201
Radiation	14 (73.7)	54 (68.4)	0.321
Death, no (%)	14 (73.7)	35 (44.3)	0.021

**Figure 1:**
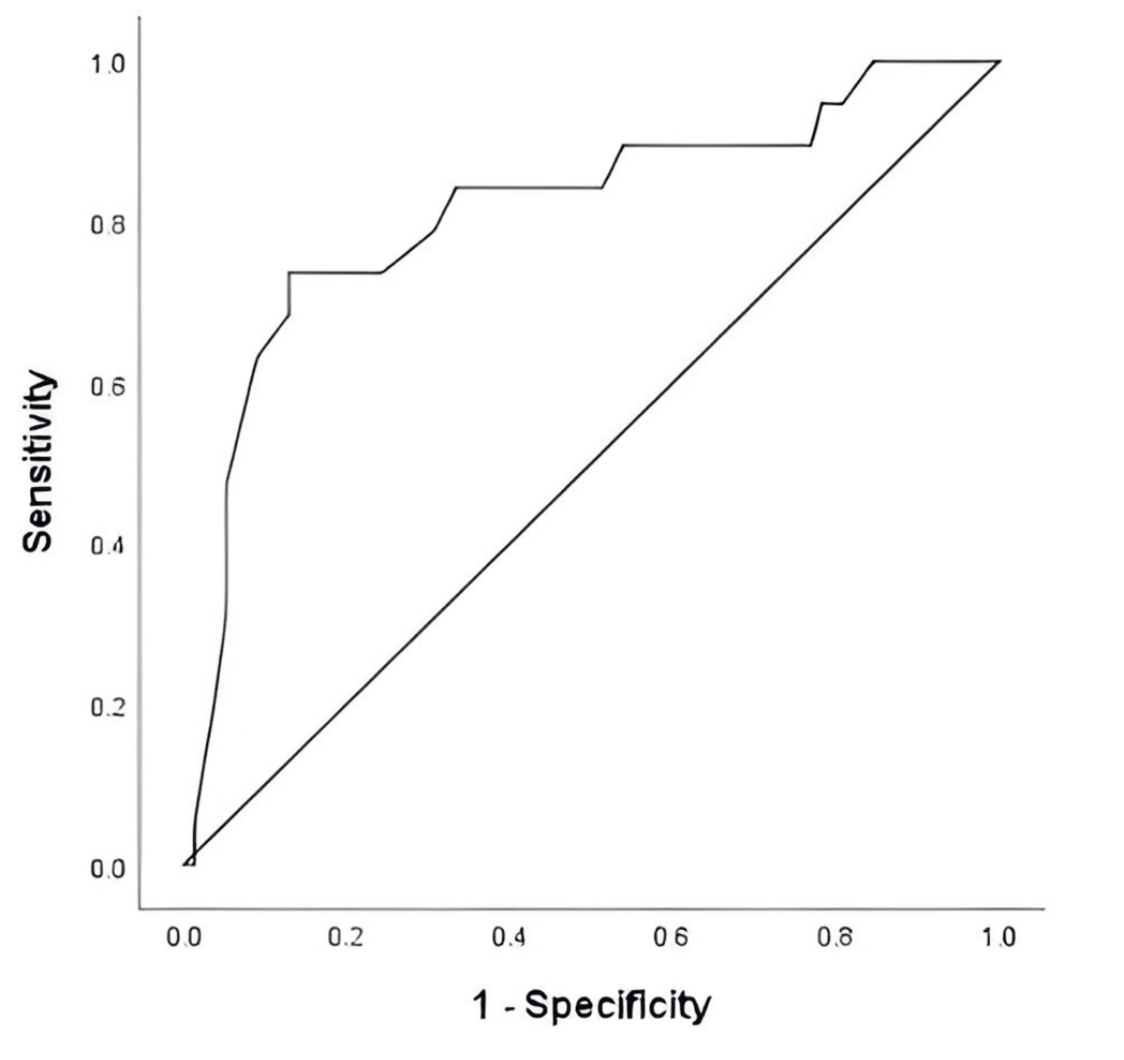
ROC curve for serum albumin level associated with PE recurrence in patients with lung cancer ROC: Receiver operating characteristic; PE: pulmonary embolism.

**Figure 2:**
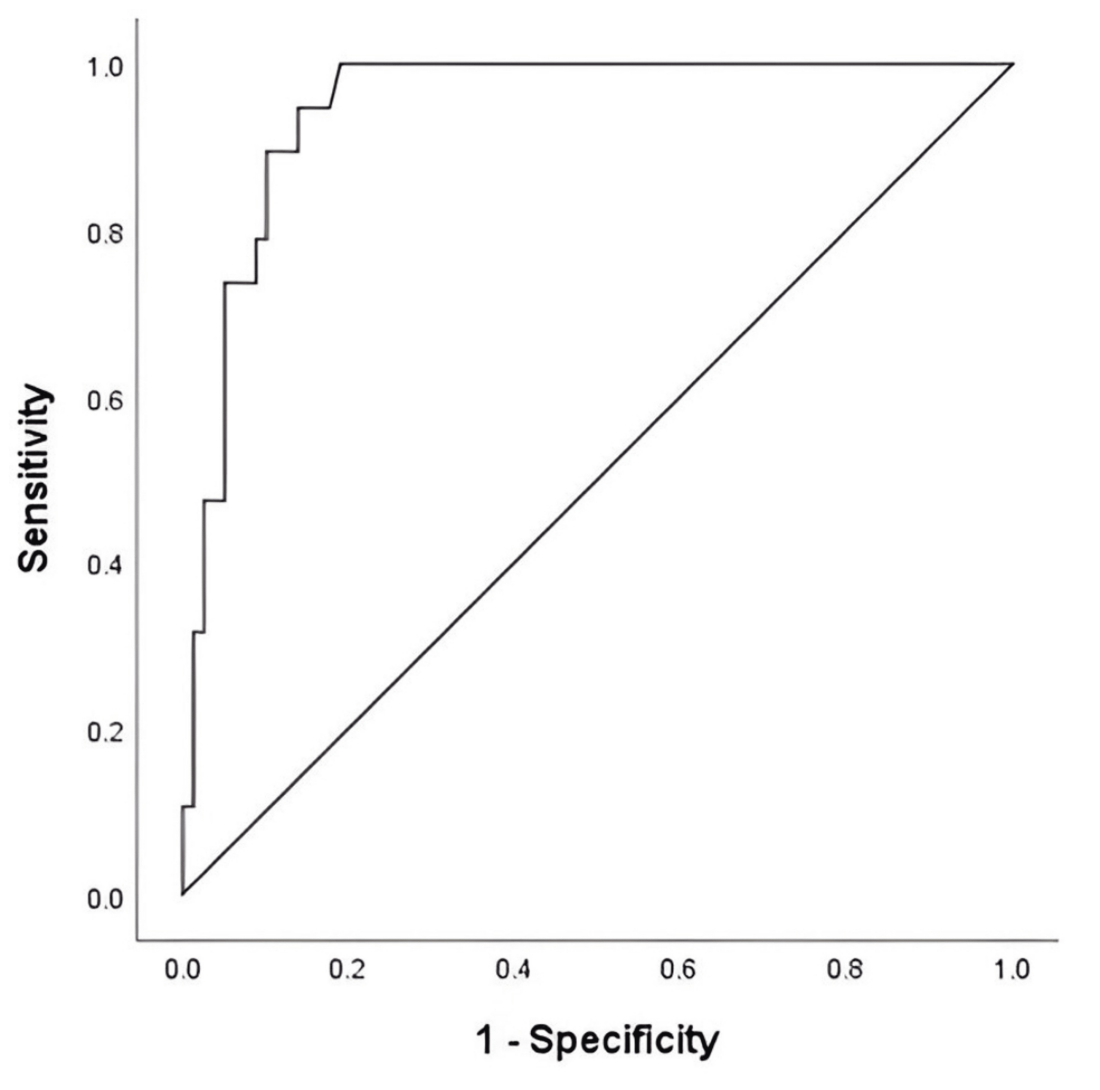
ROC curve for D-dimer associated with PE recurrence in patients with lung cancer ROC: Receiver operating characteristic; PE: pulmonary embolism.

The ROC curve analysis was performed to determine the best cutoff points of serum albumin level and D-dimer (Figures 1, 2). The highest positive likelihood ratios using the area under the ROC curve were noted when serum albumin level and D-dimer threshold of ≤ 20.5 g/L and ≥ 6.5 mg/L, respectively, were chosen. At this threshold, the serum albumin level and D-dimer were 78% and 94% sensitive, respectively, and 87% and 86% specific, respectively, in predicting recurrence of VTE. The area under the ROC curve for the serum albumin level and D-dimer was 0.815 (95% CI: 0.696 - 0.935, p < 0.001), 0.802, and 0.948 (95% CI: 0.906 - 0.989, p < 0.001), respectively. 

The detailed laboratory and pathology data are presented in Table 2. Most patients (73.7%) who developed recurrence of PE were diagnosed with adenocarcinoma compared to 40.5%who did not develop recurrence. In addition, low serum albumin level ≤ 20.5 g/L and D-dimer ≥ 6.5 mg/L were associated with the PE recurrence.

**Table 2:** Laboratory and pathology results SCLC: small cell lung cancer; NSCLC: non-small cell lung cancer.

Variables	Recurrence (n = 19)	No recurrence (n = 79)	p value
SCLC, no (%)	1 (5.3)	3 (3.8)	0.584
NSCLC, no (%)	12 (63.2)	53 (67.1)	0.745
Adenocarcinoma¸ no (%)	14 (73.7)	32 (40.5)	0.009
Squamous cell, no (%)	4 (21.1)	15 (19.0)	0.530
Metastasis, no (%)	3 (15.8)	5 (6.4)	0.187
Serum albumin ≤ 20.5 g/L	14 (73.7)	28(35.4)	0.002
D-dimer ≥ 6.5 mg/L	13 (68.4)	23 (29.1)	0.001

Univariable and multivariable logistic regression analyses for predictors associated with PE recurrence in lung cancer patients are presented in Table 3. Four out of the five variables were shown to be independent predictors of developing PE recurrence in lung cancer patients, including adenocarcinoma (OR, 2.82; 95% CI, 1.35 - 7.05; p = 0.029), systemic infection (OR, 3.96; 95% CI, 1.47 - 6.51; p = 0.043), D-dimer ≥ 6.5 mg/L (OR, 4.15; 95% CI, 1.83 - 11.85; p = 0.005), and serum albumin level ≤ 26 g/L (OR, 4.91; 95% CI, 1.64 - 12.76; p = 0.013). However, some known risk factors, such as immobility, recent surgery, long-distance travel, anticoagulation use, and cancer status at recurrence, were not consistently documented and could not be included; therefore, unmeasured confounding cannot be excluded.

**Table 3:** Univariable and multivariable logistic regression analysis for predictors associated with PE recurrence ECOG: Eastern Cooperative Oncology Group; PE: pulmonary embolism; PS: performance status.

Variables	Univariable		Multivariable	
	OR (95% CI)	p value	OR (95% CI)	p value
Poor PS (ECOG ≥ 2)	3.71 (1.22 - 11.29)	0.021	2.32 (0.60 - 6.39)	0.125
Adenocarcinoma	3.02 (1.02 - 8.77)	0.042	2.82 (1.35 - 7.05)	0.029
Systemic infection	4.82 (1.58 - 14.78)	0.006	3.96 (1.47 - 6.51)	0.043
D-dimer ≥ 6.5 mg/L	5.28 (1.79 - 15.57)	0.003	4.15 (1.83 - 11.85)	0.005
Serum albumin ≤ 20.5 g/L	5.10 (1.66 - 15.64)	0.004	4.91 (1.64 - 12.76)	0.013

Univariable and multivariable logistic regression analysis for predictors associated with death in patients with lung cancer is shown in Table 4. Two out of the four variables were found to be significant predictors associated with death in patients with lung cancer, including: adenocarcinoma (OR, 3.01; 95% CI, 1.35 - 6.85; p = 0.001), Low serum albumin level ≤ 20.5 g/L (OR, 4.51; 95% CI, 2.48 - 9.96; p = 0.001). Measures of severity of PE [such as the Pulmonary Embolism Severity Index (PESI) score] were not included in the regression model; therefore, the influence of unmeasured confounders cannot be excluded.

**Table 4:** Univariable and multivariable logistic regression analysis for predictors associated with mortality ECOG: Eastern Cooperative Oncology Group; PE: pulmonary embolism; PS: performance status.

Variables	Univariable		Multivariable	
	OR (95% CI)	p value	OR (95% CI)	p value
Poor PS (ECOG ≥ 2)	2.10 (1.02 - 5.29)	0.021	1.34 (0.50 - 4.39)	0.401
Adenocarcinoma	3.82 (1.12 - 8.77)	0.042	3.01 (1.35 - 6.85)	0.001
PE recurrence	3.52 (1.15 - 9.81)	0.027	1.48 (0.65 - 5.91)	0.431
Serum albumin ≤ 20.5 g/L	4.87 (2.66 - 11.64)	0.004	4.51 (2.48 - 9.96)	0.001

## Discussion

The aim of the current study in lung cancer patients was to identify the factors associated with PE recurrence as well as those that may influence mortality. The results showed a recurrence rate of 19.4% and a death rate of 73.7%, with an overall mortality of 50%. These findings clearly reflect the gravity of PE recurrence and highlight the need for better management strategies in these patients. In our univariable analysis, poor ECOG performance status (≥ 2) appeared to be associated with PE recurrence. This could be accounted for by the increased risk of blood clot formation in the legs as a result of decreased mobility, which in turn may worsen the overall prognosis and accordingly remains a significant concern to the clinician [11]. Two recent reports have also confirmed that poor performance status is a negative prognostic factor, resulting in poor outcomes among lung cancer patients [12,13]. However, in our study, this association did not remain significant in the multivariable logistic regression, where adenocarcinoma histology, systemic infection, low serum albumin, and elevated D-dimer were identified as independent predictors. This discrepancy is likely due to the relatively small sample size of our cohort-only 19 patients experienced recurrence-which limits statistical power and underscores the need for cautious interpretation.

Furthermore, the study revealed that recurrent PE in lung cancer patients was associated with a significantly higher occurrence of systemic infection, in line with other studies linking infections to poor prognosis in cancer and treatment-related complications [14]. Infections in this context can involve the respiratory tract, bloodstream, urinary tract, or gastrointestinal tract. They may arise directly from the cancer itself or from its treatments: lung tumors can obstruct airways leading to bronchitis or pneumonia, while chemotherapy and radiation can cause immunosuppression. The risk of infection is further increased by older age, smoking, and comorbid chronic obstructive pulmonary disease (COPD) [14].

In the current study, two additional blood factors were found to be closely linked with recurrent PE: significantly lower serum albumin and elevated D-dimer levels in patients with recurrence of PE, when compared to patients without recurrence. The decrease in serum albumin levels is a marker of poor nutritional state and systemic inflammation, both being vital in the prognosis of cancer [15-17]. In contrast, poor nutrition, other than being a serious limiting factor in oncologic treatments, has serious consequences, leading to reduced tolerance to anticancer treatment, poorer prognosis, and waste of the avoidable health care resources associated with prolonged and repeated hospitalizations [16,17]. 

On the other hand, D-dimer is a protein fragment resulting from the breakdown of fibrin or blood clot. D-dimer is released into the bloodstream and its concentration is determined by a blood test that is nowadays recognized as diagnostic of venous thromboembolism [18]. It is important to note that D-dimer is a highly dynamic biomarker, with levels influenced by the presence of a clot, active malignancy, and initiation of anticoagulation. In our study, D-dimer was measured within 24 hours of confirmed PE, but the exact timing was not standardized. These factors should be considered when interpreting its predictive value for PE recurrence, as also highlighted in previous studies [19]. The elevated blood D-dimer level and low serum albumin have also been validated to predict the PE recurrence in cancer patients [20-23]. The results of the current study have shown that both elevated D-dimer as well as low serum albumin can serve as satisfactory predictors of recurrence of PE. The association between low serum albumin level and thrombosis was explained by the fact that albumin is known to have both heparin-like antithrombotic activity as well as being an inhibitor of platelet aggregation; loss of these functions underlies the increased tendency to thrombosis [24]. 

In the present study, the mortality rate among lung cancer patients with recurrence of PE was 73.7%, while the overall mortality rate for the cohort was 50%. To find the possible predictors for mortality, we employed multivariable logistic regression analysis, and the results revealed two independent predictors: adenocarcinoma and low serum albumin. The lesson to take from the findings of this study is the need for regular monitoring of serum albumin and D-dimer levels in lung cancer patients who have PE. In fact, a recent systematic review and meta-analysis identified D-dimer level as an independent predictor of poor prognosis in patients with lung cancer; high plasma D-dimer level leads to lower survival than in the low D-dimer level [25].

The strengths of this study include a well-defined cohort of patients with lung cancer and prior PE, sophisticated statistical methods for factor identification, and the evaluation of several biomarkers for predicting PE recurrence and mortality. However, the study is limited by its retrospective, single-center design and relatively small sample size. Some potentially relevant clinical variables, such as cancer stage, disease status, and detailed PE management strategies, were not available and may have influenced outcomes. In addition, residual confounding cannot be fully excluded. Therefore, the recurrence and mortality rates reported should be interpreted within the context of this study cohort. Future prospective, multi-center studies are needed to validate these findings and to explore additional biomarkers and interventional strategies. Such efforts will further advance understanding of risk factors and optimize management of PE in lung cancer patients.

## Conclusions

This study emphasizes the enormous difficulties encountered by patients with lung cancer and PE. PE has a 19.4% recurrence rate and a 73.7% mortality rate among those with lung cancer and recurrence of PE. The main predictors of PE recurrence are adenocarcinoma, systemic infection, elevated D-dimer levels, and low serum albumin levels; adenocarcinoma and low serum albumin levels predicted mortality. Therefore, our findings suggest that high D-dimer and low serum albumin are associated with an increased risk of PE recurrence in patients with lung adenocarcinoma. Nonetheless, given the retrospective, single-center design and small sample size of this study, these results should be interpreted with caution. Larger, prospective multi-center trials are needed to validate these predictors and guide clinical decision-making in managing lung cancer patients with PE.

## References

[1] Lyman GH, Culakova E, Poniewierski MS, Kuderer NM (2018). Morbidity, mortality and costs associated with venous thromboembolism in hospitalized patients with cancer. Thromb Res.

[2] Yang R, Wang H, Liu D, Li W (2024). Incidence and risk factors of VTE in lung cancer: a meta-analysis. Ann Med.

[3] Li Y, Shang Y, Wang W, Ning S, Chen H (2018). Lung cancer and pulmonary embolism: what is the relationship? A review. J Cancer.

[4] Kumar DR, Hanlin E, Glurich I, Mazza JJ, Yale SH (2010). Virchow's contribution to the understanding of thrombosis and cellular biology. Clin Med Res.

[5] Koizume S, Miyagi Y (2022). Tissue factor in cancer-associated thromboembolism: possible mechanisms and clinical applications. Br J Cancer.

[6] Ahmadi SE, Shabannezhad A, Kahrizi A (2023). Tissue factor (coagulation factor III): a potential double-edge molecule to be targeted and re-targeted toward cancer. Biomark Res.

[7] Wolberg AS, Campbell RA (2008). Thrombin generation, fibrin clot formation and hemostasis. Transfus Apher Sci.

[8] Reddel CJ, Tan CW, Chen VM (2019). Thrombin generation and cancer: contributors and consequences. Cancers (Basel).

[9] West HJ, Jin JO (2015). JAMA oncology patient page. Performance status in patients with cancer. JAMA Oncol.

[10] Oken MM, Creech RH, Tormey DC (1982). Toxicity and response criteria of the Eastern Cooperative Oncology Group. Am J Clin Oncol.

[11] Farmakis IT, Barco S, Mavromanoli AC, Konstantinides SV, Valerio L (2022). Performance status and long-term outcomes in cancer-associated pulmonary embolism: insights from the hokusai-VTE cancer study. JACC CardioOncol.

[12] Friedlaender A, Liu SV, Passaro A, Metro G, Banna G, Addeo A (2020). The role of performance status in small-cell lung cancer in the era of immune checkpoint inhibitors. Clin Lung Cancer.

[13] Sehgal K, Gill RR, Widick P (2021). Association of performance status with survival in patients with advanced non-small cell lung cancer treated with pembrolizumab monotherapy. JAMA Netw Open.

[14] Budisan L, Zanoaga O, Braicu C (2021). Links between Infections, lung cancer, and the immune system. Int J Mol Sci.

[15] Cordeiro LAF, Silva TH, de Oliveira LC, Neto JFN (2020). Systemic inflammation and nutritional status in patients on palliative cancer care: a systematic review of observational studies. Am J Hosp Palliat Care.

[16] Caccialanza R, Cotogni P, Cereda E (2022). Nutritional support in cancer patients: update of the italian intersociety working group practical recommendations. J Cancer.

[17] Da Prat V, Pedrazzoli P, Caccialanza R (2024). Nutritional care for cancer patients: are we doing enough?. Front Nutr.

[18] Khan F, Tritschler T, Kahn SR, Rodger MA (2021). Venous thromboembolism. Lancet.

[19] Posch F, Riedl J, Reitter EM (2020). Dynamic assessment of venous thromboembolism risk in patients with cancer by longitudinal D-Dimer analysis: a prospective study. J Thromb Haemost.

[20] Al-Eidan FAS, Alotaibi SA, Almajid HM, Alnahedh TA, Abdel Gadir AG (2023). High D-dimer level at first incident cancer-associated venous thromboembolism is a predictor for recurrence: a retrospective cohort study. J Appl Hematol.

[21] Aleidan FAS, Almesfir R, Alqudaibi N, Alqhatani S, Abuelgasim KA (2024). Incidence and predictors of venous thromboembolism and mortality in Saudi lung cancer patients: a retrospective two-center cohort study. J Appl Hematol.

[22] Stevens H, Peter K, Tran H, McFadyen J (2020). Predicting the risk of recurrent venous thromboembolism: current challenges and future opportunities. J Clin Med.

[23] Basili S, Carnevale R, Nocella C (2019). Serum albumin is inversely associated with portal vein thrombosis in cirrhosis. Hepatol Commun.

[24] Valeriani E, Pannunzio A, Palumbo IM (2024). Risk of venous thromboembolism and arterial events in patients with hypoalbuminemia: a comprehensive meta-analysis of more than 2 million patients. J Thromb Haemost.

[25] Ma M, Cao R, Wang W (2021). The D-dimer level predicts the prognosis in patients with lung cancer: a systematic review and meta-analysis. J Cardiothorac Surg.

